# Theoretical-methodological essay on gender identity information validity in epidemiological surveillance of violence

**DOI:** 10.1590/1980-220X-REEUSP-2023-0240en

**Published:** 2023-10-20

**Authors:** Ricardo de Mattos Russo Rafael, Adriana Costa Gil, Helena Gonçalves de Souza Santos, Jaime Alonso Caravaca-Morera, Karen Lucas Breda

**Affiliations:** 1 Universidade do Estado do Rio de Janeiro, Faculdade de Enfermagem, Departamento de Enfermagem de Saúde Pública, Rio de Janeiro, RJ, Brazil.; 2 Universidade do Estado do Rio de Janeiro, Faculdade de Enfermagem, Rio de Janeiro, RJ, Brazil.; 3 Universidad de Costa Rica, San José, Costa Rica.; 4 University of Hartford, College of Education, Nursing & Health Professions, Department of Nursing, West Hartford, Connecticut, United States of America.

**Keywords:** Gender Diversity, Gender Identity, Violence, Epidemiological Monitoring, Public Health, Diversidad de Género, Identidad de Género, Violencia, Monitoreo Epidemiológico, Salud Pública, Diversidade de Gênero, Identidade de Gênero, Violência, Monitoramento Epidemiológico, Saúde Pública

## Abstract

The inclusion of the “gender identity” field in the Brazilian violence surveillance system, although representing a step forward, still has limitations that may compromise epidemiological data validity. Existing response options for victims’ identities do not adequately cover the diversity of this analysis category, resulting in classification biases. Additionally, the absence of options for cisgender identities reflects an approach that naturalizes these identities, while trans identities are considered deviant and subject to surveillance. To overcome these limitations, it is imperative to adopt a broader understanding of gender as a social and performative construction. This requires a reassessment of social structures and data collection instruments. In this context of discussion, this theoretical-methodological essay aims to reflect on gender identity measurement in the Reporting Diseases System interpersonal and self-inflicted violence surveillance system, taking as frameworks the theoretical conceptions about gender as a performative act and the foundations of validity in epidemiological investigations.

## RESUMO

A inclusão do campo “identidade de gênero” no sistema de vigilância de violências brasileiro, embora tenha representando um avanço, ainda apresenta limitações que podem comprometer a validade dos dados epidemiológicos. As opções de resposta existentes para as identidades das vítimas não abrangem adequadamente a diversidade dessa categoria de análise, resultando em vieses de classificação. Adicionalmente, a ausência de opções para as identidades cisgênero reflete uma abordagem que naturaliza essas identidades, enquanto as identidades trans são consideradas desviantes e passíveis de monitoramento. Para superar essas limitações, é imprescindível adotar uma compreensão mais ampla do gênero como uma construção social e performativa. Isso requer uma reavaliação das estruturas sociais e dos instrumentos de coleta de dados. Nesse contexto de discussão, este ensaio teórico-metodológico tem como objetivo refletir sobre a aferição da identidade de gênero no sistema de vigilância de violências interpessoais e autoprovocadas do Sistema de Agravos de Notificação, tomando como referenciais as concepções teóricas sobre gênero como ato performativo e os fundamentos da validade em investigações epidemiológicas.

## RESUMEN

La inclusión del campo de la “identidad de género” en el sistema brasileño de vigilancia de la violencia, aunque representa un paso adelante, todavía tiene limitaciones que pueden comprometer la validez de los datos epidemiológicos. Las opciones de respuesta existentes para las identidades de las víctimas no cubren adecuadamente la diversidad de esta categoría de análisis, lo que genera sesgos de clasificación. Además, la ausencia de opciones para las identidades cisgénero refleja un enfoque que naturaliza estas identidades, mientras que las identidades trans se consideran desviadas y sujetas a seguimiento. Para superar estas limitaciones, es imperativo adoptar una comprensión más amplia del género como una construcción social y performativa. Esto requiere una reevaluación de las estructuras sociales y de los instrumentos de recolección de datos. En este contexto de discusión, este ensayo teórico-metodológico tiene como objetivo reflexionar sobre la medición de la identidad de género en el sistema de vigilancia de la violencia interpersonal y autoinfligida del Sistema de Enfermedades de Declaración Obligatoria, tomando como referentes las concepciones teóricas sobre el género como acto performativo y los fundamentos de validez en las investigaciones epidemiológicas.

## INTRODUCTION

Violence is a socio-historical, complex and comprehensive phenomenon that negatively affects both the physical and psychosocial health of victims and their families^(1)^. Recognizing the urgent need to monitor and scrutinize violence as a+ public health phenomenon in Brazil, in 2006 the Ministry of Health implemented the Violence and Accident Surveillance System (VIVA - *Sistema de Vigilância de Violências e Acidentes*), linked to the Reporting Diseases Information System (SINAN - *Sistema de Informação de Agravos de Reporting*) from 2009. Reporting cases of interpersonal and self-inflicted violence has become mandatory for health services across the country since 2011^(2)^.

By making reporting compulsory, the Ministry of Health sought to create a comprehensive and up-to-date database on the incidence of cases of violence, producing essential information to support public policies aimed at tackling this phenomenon, in addition to allowing identifying more vulnerable groups and regions. Since then, several improvements have been made to the VIVA system report forms, and the last one, carried out in 2014 and implemented in 2015, was one of the most important. Driven by the Brazilian National LGBTQIA+ Comprehensive Health Policy (*Política Nacional de Saúde Integral LGBTQIA+*) and by pressure from social movements, this update included fields for recording victims’ orientation and gender identity^(2,3)^.

This inclusion, therefore, formally recognized gender and sexual diversity markers as essential elements in the social determinants of health, illness and death. By including fields for recording the gender identity and sexual orientation of victims of violence, it became possible to identify and monitor cases against LGBTQIA+ people, representing an indisputable advance in the epidemiological surveillance of the phenomenon^(3,4)^.

Not by chance, from 2015 to 2017, there was a significant increase in interpersonal and self-inflicted violence reports, both among the LGBTQIA+ community and among travestis and transsexuals (in this text, the term “trans” will be adopted in respect to the political movement transactivist). The quality of data on sexual orientation and gender identity over the period has improved significantly over the years, with physical and sexual forms of violence being the most observed in reports^(3)^. However, this advance should not represent the end of the debate on the matter, especially when analyzing that the inclusion of fields and format of sexual and gender diversity markers did not occur from a comprehensive understanding of the topic.

Taking the “sexual orientation” field of the report form^(2)^ as a reference, it is possible to observe that this variable has response options that include a self-determination of being heterosexual, homosexual (gay/lesbian), bisexual, does not apply and ignored, closing the possibility of other and multiple forms of sexual orientation, such as queer, intersex, pansexual, asexual and other orientations, represented by the “plus” symbol in the acronym LGBTQIA+. Even more restrictively, the gender identity variable included only response options for “travesti”, “transsexual woman”, “transsexual man”, “does not apply” and “ignored”.

The lack of options for responses such as “cisgender woman”, “cisgender man” and other identities, including those that self-determine as gender fluid, queer, among others, entails significant problems in terms of epidemiological validity. This is because options “does not apply” or “ignored” can be assigned both to cisgender people and to other identities that do not fit into these pre-established categories, resulting in classification biases that are difficult to control during analyses. It is crucial to reflect on the fact that mapping projects, surveying, and producing epidemiological data are important technologies for the population’s acknowledgment and public policy formulation^(4,5)^. However, it is equally urgent that data production evolves towards more sophisticated information standards in order to mitigate the damage that restrictive classification can cause.

Moreover, the lack of adequate options for autonomous gender identification and therefore the worthiness of people establishes a discursive effect in the official reporting document, leading to an epidemiological mapping that focuses only on genders considered deviant from the norm. To wit, as cisgender categories are ignored, there is the establishment of the positive – the abnormal, what needs to be seen and surveilled – only for the *travesti* and transgender population. This reflects an approach that is commonly seen in disease mapping, where the focus is to mark positive cases and establish a standard of normality for the majority of the population. In other words, the discourse underlying the form suggests that cisgender identities do not need to be mapped. Thus, by ignoring these identities, the form considers them as natural, adding a meaning of originality to cisgender identities and a pathological or deviant meaning to travesti and trans identities as well as ignoring and erasing non-binary, intersex and other non-cisgender identities.

This manuscript draws upon Judith Butler’s theories^(6-8)^ to comprehensively understand the social construction of gender and to question the fundamental norms and categories that limit and oppress people based on their gender identity. By viewing gender as a social and consequently performative construct, the authors take a theoretical stance to challenge and question the hegemonic perspective that perceives gender as a fixed and essentialist category (biological and innate). These challenges the notions rooted in binary thinking that systematically exclude and erase certain identities within various social structures. Consequently, this erasure hinders the provision of care and the formulation of public policies capable of recognizing and addressing issues faced by socially considered dissonant groups.

In this context, this theoretical-methodological essay aims to reflect on the measurement of gender identity in the SINAN surveillance system for interpersonal and self-inflicted violence, taking as frameworks the theoretical conceptions about gender as a performative act and the foundations of validity in epidemiological investigations.

### Subversion of the Concepts of Sex and Gender Originality: a Debate on the Effects of Power, Discourse and Performativity 

For a long time, the process of biodeterministic differentiation of sexes, based on theories that attribute body anatomy and genetics as an explanation, played a significant role in building the social roles traditionally assigned to men and women. For a significant period, the concept of humanity being divided into two distinct categories based on sexual differences has long been considered an axiom. Furthermore, in these currents guided by the biological essentialism rationale, the categories “sex” and “gender” were often used interchangeably^(9,10)^. In this approach, female bodies were often interpreted as an inferior version of male bodies, which were considered the original standard, with no room for other gender identities^(10)^. This line of thought has long used this effort to explain potential inequalities between men and women. This line of thought has long used - and still uses - this rhetorical effort to explain inequalities between men and women.

However, in her seminal work, Beauvoir^(11)^ makes a scathing critique of this view, an aspect that even today there are still those who consider it subversive and dangerous^(12)^, since she questions the process of stabilizing gender as roles to be developed in society. Beauvoir^(11)^ inaugurates a new line of thought by interpreting gender as a socio-anthropological construction and, consequently, culturally disconnected from the supposed biological differences between sexes. Thus, the reductionist view practiced so far is contested, and paves the way for a more comprehensive and inclusive understanding of expressions of gender.

To the same extent, authors such as Betty Friedan^(13)^, bell hooks^(14)^, among many others, walked in the same direction, endorsing the struggle of the feminist movement to contest the roles and social inequalities imposed by gender, in addition to, some of them, including race and class as important mediators of the state of inequalities imposed by society. In her widely recognized work in the field of gender studies, entitled “Gender: A Useful Category of Historical Analysis”, Joan Scott^(15)^ aligns herself with current socio-anthropological currents as well as assumes influences from post-structuralist theories. In this context, Scott challenges the dichotomy between the essentialist nature of men and women, proposing a perspective that assigns a crucial role to language and discourse in gender formation.

Scott^(15)^ argues that gender is not an innate characteristic, but rather a social construction that is shaped and perpetuated through symbolic representations. For her, gender should not be treated as a fixed category, but should be regarded as a challenge to the meanings attributed to the differences between men and women, particularly questioning the exclusion of certain groups in favor of others considered hegemonic^(10)^. In this perspective, this thought is combined with Beauvoir’s famous phrase: “One is not born a woman, but becomes one”^(11)^.

On the other hand, even if this perspective places the focus on the cultural context that shapes and builds meanings to bodies and genders, even so, bodies, ultimately, would be doomed to the molds of culture, becoming the new destiny of being^(8)^. Therefore, even if other destinations for bodies are questioned and a destiny initially traced by what is biological is summarily replaced and rejected, the stability of destiny would remain, but this time through culture^(8,16)^. As Firmino and Porchat^(16)^ point out, this process of shaping the female gender would encounter societal constraints and norms (even if implicit), imposing a new imperative on the body: “become a woman!”^(16:56)^.

Clearly, this set of rules about what is feminine would be subject to a limited and socially acceptable definition of what it means to be a woman^(12)^. In other words, the construction of the feminine does not escape the weight of cultural expectations, even if it can resist biological determinism, bringing with it a set of characteristics that would be considered the original version of being a woman^(8,12,16)^. Deviation from these established norms can be seen as subversion or erratic behavior and consequently is subject to social corrections. In fact, social constraints occur and in some bodies considered dissonant, even sexual coercion, such as rape with the aim of “correction” and “cure”, perpetrated against lesbian women and trans men^(17)^.

In this understanding, even male identity, which in the past benefited from the status of an original category, would not be exempt from this process of becoming and, consequently, from what is culturally necessary to gather in terms of male characteristics and behaviors. Consequently, the theoretical understanding of the separation of what is biological, sex, from what would be a category constructed by the discourse that stabilizes within a culture, gender, is questioned^(8,10,12,16,18)^.

Thus, from the moment people are identified as women or men, or, from a biological perspective, male and female, they become inscribed in a system of social norms^(8,12,16)^. In this vein, the detection of sex, even in an unborn body, becomes affected by discourses that outline expectations about desires, behaviors, and appearances based on the slightest detection of what is or what will possibly become the genitalia of that body^(16)^. Actually, even before the possibility of detecting the genitalia, a set of familial and social expectations already impacts the body. These expectations will confine the as-yet-unborn body to a sort of path of expected behaviors taught by adults through multiple messages throughout life. Therefore, social rules, through a pre-existing discourse, although not necessarily deterministic and fateful, help shape and restrict certain experiences, desires, and, consequently, gender expressions, as well as contribute to the illness of individuals^(18)^.

Therefore, John L. Austin’s philosophy^(19)^, which, like Foucault’s works^(20,21)^, supports Butler’s theory, brings up the argument that discourse is not intended merely to describe the world, meaning that language is not always merely constative. For him, language can also be used to produce actions through words that are used in relation to what he would conceptualize as fundamental units, which go beyond word meaning, being in fact speech acts.

By producing actions and effects in the context in which language is used, Austin^(19)^ states that we are dealing with performative language. In this regard, discourse exhibits fundamental characteristics that encompass conventions, intentions, and acts capable of impacting the world. The first characteristic revolves around the recognition that the execution of a speech act is contingent upon the surrounding context and established norms. Put differently, within the realm of communication, there must exist an implicit agreement concerning word meanings within a given context. Consequently, the use of words and expressions is not arbitrary; rather, their meanings, within historical and contextual parameters, are predefined even before the onset of a dialogue.

The second characteristic is intertwined with the concrete intention of effecting an action while conveying a message, whether through the written or spoken word. In this context, transmitting a message is not solely an expression of ideas or the assertion of facts. Language can also be an intentional act with a purpose underlying the message itself. Finally, the third characteristic, the perlocutionary act, pertains to the impact generated by the act of speech, i.e., the consequences induced by speakers on the world and their audience. Beyond mere information dissemination, speech can function as a catalyst for actions capable of influencing individuals and eliciting reactions.

Thus, when contemplating these characteristics of language as performative, Butler^(7,8)^ connects with Austin’s proposal^(19)^. Consequently, language is not merely a neutral tool of communication; it also serves as a form of action that shapes social interactions and upholds certain gender identities that seem to be stabilized in discourse^(8)^. Consequently, it can also be used to deny diversity.

In investigating the nature of gender in this way, one is invited to examine not only the biological foundations assigned to men and women but mainly how discourse and culture operate in modulating the experience of being male or female and the strict boundaries imposed on each of these identities^(12,16)^. In the specific case of this manuscript, through discourse, it is reflected that it is possible to maintain a status of original gender identities using a specific language (e.g., terms, words) in a violence report form. Thus, meaning is attributed to what is recognizably different (“queer”) to be marked in the form (as in the case of travestis, trans men and trans women), while gender identities considered socially “original” remain strictly protected.

It is useful to reflect on the concept of originality and the process of imitation when we approach issues of gender identity according to Butler^(7,8)^, as this contributes to understanding the perverse logic of a system that seeks to correct or eliminate bodies that deviate from norms. In this system, a gender-correct and, therefore, original relationship is established throughout history and through performative processes. Sex and gender are thus imitated over time, giving the appearance of something natural^(10,16)^. This perspective suggests that performative practices and repeated gestures help to create the notion of an inner essence of gender. Thus, when speaking of imitation, Butler^(7,8)^ does not just refer to copying of forms, but to the discursive process by which people learn and repeat those forms, behaviors, and social roles that are expected, corroborating similar remarks by Scott^(15)^.

When analyzing cisgender women, it is clear that the construction of what is considered female acts is not something essentialist, but rather the result of social and cultural influences that shape femininity from before birth, at the moment of discovery and gender expectations. This also applies to trans women, whose identities tend to be shaped by the patterns and expressions associated with what is considered female in society^(16)^. Likewise, cisgender men, trans men and other expressions of masculinity are culturally shaped and reproduced as well. The male is also a convention, a becoming^(11)^ without necessarily being born^(7,8)^. Both cisgender and transgender individuals are equally involved in the performative construction of feminine and masculine genders^(16)^. Meanwhile, those who identify as gender fluid impose an existential questioning of the binary promoted by cisgender and transgender individuals.

Therefore, the process of repeating what would be considered acts and manners of what is socially considered female or male is what Butler calls performance^(8)^. Understanding sex and gender as performances is, in itself, a questioning of the fallacious attempt to determine gender based on sex. To wit, these are not innate characteristics, but rather a production resulting from discourses and languages reproduced and stabilized in a culture over time.

Gender is not an ontological characteristic of beings, as from a continuous process of creation and recreation, is shaped by social interactions and power relations^(10)^. Butler’s main legacy is to understand gender as performative and seek the subversion of essentialist order, allowing freedom of expression and aiming at people’s gender self-determination^(7,8,10,16)^. However, after so many theoretical advances, there is no room for naivety. The hegemonic and heterocisnormative culture, in order to maintain the coherence of a dual gender system, favors some gender identities over others^(18)^.

Thus, despite the undeniable progress achieved in 2015, the interpersonal and self-inflicted violence report form is an example of the effects of performative discourse on identity construction, as it erases some gender expressions from its classification system. Ultimately, the question remains whether some identities matter more than others from an epidemiological surveillance perspective. Thus, due to the very nature of the phenomenon of violence, restricting data collection on this variable to a limited set of responses, which traditionally excludes identities in society, raises the question whether the reporting system itself would not be reproducing violations of rights in some population groups. Furthermore, these options greatly restrict surveillance measurement validity.

### Hegemonic Performative Discourse on Epidemiological Validity: the Sacrifice of the Method and Problem Strategy to Classify Groups 

Epidemiological validity is a central theme in scientific discussions, as there is a constant concern to reduce biases, random errors and problems related to the sources of information in investigations. Validity refers to the ability of a survey, its instruments or variables to correctly and accurately measure what it is intended to measure^(22)^. On this path, for a measure to be effectively valid, there must be a theoretical framework that underlies what it is intended to measure. In other words, validity does not respond to a mere metric, but, among other pillars, to an operationalization of a construct that by nature is theoretical, in a reduction capable of making it measurable^(23)^.

From a statistical point of view, validity is considered a measure of accuracy (Ac), which is the proportion of correct results, true positive (TP) and true negative (TN) in relation to the total number of results obtained. The closer to 100% the accuracy, the more accurate the measurement^(22,24)^.

$$Ac=\frac{\text{TP}}{\text{TP+TN}}\times 100$$

Accuracy measure decomposition is also key to understanding test properties (e.g., a procedure, item, or question), known as “sensitivity” and “specificity”. While sensitivity is a statistical measure that assesses the ability of a test to correctly identify positive cases for a given condition assessed, i.e., the proportion of TP in relation to the total number of positive cases identified, specificity is a measure that is concerned with correctly identify the negative cases (TN) among tests applied in the studied population. In other words, specificity identifies the proportion of correctly classified TN in relation to the total number of negative cases. The results of both properties are expressed as proportions ranging from 0 to 100, where 100 represents a degree of excellence obtained by measurement, indicating that all TP or TN were correctly classified^(24)^.

$$\begin{array}{cc} S=\frac{\text{TP}}{\text{TP+FN}}\times 100 & E=\frac{\text{TN}}{\text{TN+FP}}\times 100 \end{array}$$

Thus, considering the use of an instrument (interpersonal and self-inflicted violence report form) that only allows marking travesti identities, trans women and trans men for the purpose of investigating an identity condition with a wide variety of classifications in addition to those presented, significant challenges certainly arise with regard to epidemiological validity. Although the selected cases that self-declare as non-cisgender identities (travestis, trans men and trans women), in theory, are better identified, problems arise when dealing with cases of cisgender people. This is because this set of identities is marked as “ignored” or “does not apply” in the collection instrument. Furthermore, other non-cisgender identities (non-binary, gender fluid, asexual, etc.) are not even considered in the instrument.

Although the instrument manages to detect with relative adequacy the selected cases of non-cisgender identity, the presence of options “ignored” and “does not apply” introduces a factor of uncertainty in measurement. These cases may include individuals who test positive for undisclosed identities, such as travesti and transgender people who, for various reasons, chose not to disclose their identity. This may result in an underestimation of instrument sensitivity. Another crucial aspect is instrument specificity. Since negative cases are not marked as such, there is a lack of accurate identification of TN. This impairs epidemiological validity, as it is not possible to correctly distinguish negative cases from ignored cases, potentially overestimating cisgender condition prevalence. To wit, the absence of accurate reporting for cases of violence against cisgender people makes it difficult to compare groups, reducing the ability to diagnose the situation and formulate public policies aimed at the country’s real needs.

One way of dealing with the validity and comparability problems associated with collecting the “gender identity” item on the report form is to establish a relationship between the gender variable - although, as discussed in the previous section, this is far from ideal from the point of view of gender studies - and gender identity. In this sense, the male response as “does not apply” in gender identity would be equivalent to a cisgender man. Likewise, when sex is registered as “female” and gender identity is “does not apply”, it is a cisgender woman^(2)^. However, as noted, this does not solve all the presented validity problems.

Although it may seem like a methodologically simple solution to be carried out, the question remains as to how much measurement instruments can be alien to life’s concrete reality as well as how much they can serve to maintain a state that produces inequality and annulment of gender identities through the use of a performative language^(19)^ that reinforces the power^(20)^ over the originality of some genders^(7,8)^. The introduction of response options “cisgender woman” and “cisgender man” would address this problem imposed by a collection instrument, although it would not resolve all reality identification situations.

The introduction of a field with “other identities” would greatly contribute to the epidemiological investigation process, an aspect absent until the present version of the form. It is noted that people who identify as gender fluid, in the absence of option “others”, tend to naturally choose “does not apply” in their gender identity. However, if they have their gender assigned at birth as “male”, they would be classified as “cisgender men”, a clear epidemiological classification bias. This, obviously, does not represent the identity reality to be identified, introducing analytical problems that are difficult to control.

Another situation that illustrates a potential classification bias in the current formatting of sex and gender variables is the fact that a trans woman who, after the struggle to rectify her civil registry, will have a female sex record in her documentation. If she is not questioned about her gender identity, and this depends on how she is questioned, gender identity classification will tend to be “ignored” or “does not apply”, generating the understanding of a cisgender woman.

Ultimately, the problem that arises from the form’s poor classification, in addition to the underestimations reported above, reinforces the language of ambiguity between sex and gender so criticized by current theories^(6–8)^. In other words, the form, in addition to an in-depth analysis of its gains with the inclusion of these variables, reproduces a hegemonic and performative language^(19)^, and reinforces the sense of originality of some identities over others. Additionally, the current form configuration form imputes the erasure of gender fluid people, intersex people and a huge variety of gender identities, even sacrificing sensitivity as a test property^(22,24)^.

The existence of people who challenge the socially imagined relationship between sex and gender further illustrates the not merely biological nature of gender identities and the classification problems imposed by formatting this variable on the form. By demonstrating that the relationship between sex and gender is more complex than it appears^(8,16)^, intersex people naturally embarrass conventionally established binary definitions. The same occurs with people with a gender-fluid identity, whose existence questions the alleged originality of gender and sexual orientation, revealing the gap between what one intends to grasp and the concrete and complex reality of diverse bodies in the world. In the current version of the report form, there is no space for expressing and gathering information about these identities, eliminating the sexual and gender diversity of these population groups.

**Chart 1:**
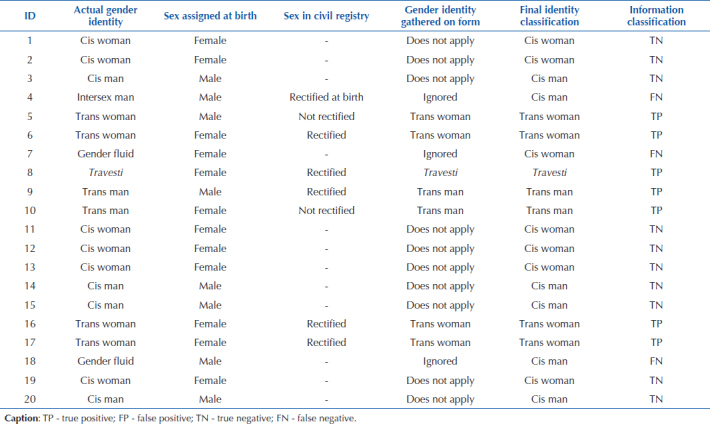
Hypothetical database with 20 reporting records of interpersonal and self-inflicted violence containing the variables gender identity, sex assigned at birth, sex in civil registry, gender identity gathered in form, identity classification and information classification – Rio de Janeiro, RJ, Brazil, 2023.

Taking a hypothetical population with 20 records as a reference (Chart 1), the issue of epidemiological validity can be better illustrated. In the chart, it is possible to verify actual gender identity, the one that is self-reported and that is intended to be identified in the form, sex assigned at birth, as information identified through self-reporting or, more generally, through reported people’s civil record, current status of sex in civil registry (whether it has been rectified or not), and gender identity, identified by checking the options in the report form. From this, the exercise of classifying (“final classification”) gender identity is carried out from reported sex and gender identity gathered in the form with the aim of generating a database aimed at comparability between groups. Subsequently, the information was classified as “TP” and “TN”, and as “false positive” and “negative”.

From this simple exercise, it is observed that, with a specificity of 100% for the hypothetical population, it is possible to infer that the test was able to correctly identify all truly negative cases, i.e., all cisgender people were correctly classified. The form’s relative efficiency in this hypothetical exercise was also observed in relation to the sensitivity property, being possible to correctly identify 70% of TP; in this case, travesti, trans woman, and trans man identities. On the other hand, the properties of the test observed in this exercise tend not to be repeated in the face of concrete reality.

It is essential to consider that not all people will answer about their gender identity, either because of fear or stigma for revealing their identity, or because they will not even be asked about it. Assuming further that, in record 6 (ID 6), if a trans woman is not questioned about her gender identity and her sex has been rectified in civil registry to female, she will be classified as a cisgender woman. This is just another example of the instrument’s reduced sensitivity in the concrete reality of everyday life. Thus, the sensitivity in the hypothetical exercise would change from 70% to 60%. Thus, it is not improbable that TP cases are misclassified as negative (FN) in this information classification process by the instrument.

A study that investigated notifications between 2015 and 2017 showed that, although there was a significant improvement in filling out “gender identities” and “sexual orientations”, respectively, 37.8% and 30.8% of responses regarding these variables were not considered valid. In other words, a significant part of the answers was ignored, demonstrating the serious problems in filling and classifying this item^(3)^. Furthermore, it is essential to reflect that, in Brazil, the variable “gender identity” is not even obligatorily collected in children under 10 years of age^(4)^, which reinforces gender expression denial since childhood, generating additional problems for the epidemiological surveillance of violence in this group. When considering this reality, the result is an increase in the number of travestis and trans people not correctly classified, greatly affecting instrument sensitivity.

By incorrectly expanding the classification of people who are exposed to greater risks of violence^(25,26,27,28)^ in the group of “ignored” and “does not apply”, which we assume here as cisgender people, there is a potential and false increase in violence prevalence in this group. This means that, when comparing cisgender groups with the group of travesti and transgender people, the difference in the magnitude of the events will be smaller than in reality, due to a measurement bias. In other words, the comparison of information with groups of cisgender people ends up being difficult due to the present bias, which impairs the validity of this information. Thus, the form’s current configuration, even with validity problems, allows for consistent assessment (i.e., according to epidemiology, reproducibly) of interpersonal and self-inflicted violence in travestis, trans women, and trans men over time. On the other hand, the form excludes other identities from surveillance and makes it difficult to establish comparability with the cisgender group.

In a country where violence is a serious public health problem and which, alarmingly, leads the ranking of murders of trans people in the world^(29)^, a completely preventable information bias like this deserves to be tackled urgently, especially since actions in the field of public health in Brazil are based on information. The less the needs of the population are identified, the lower the chances of achieving success in formulating actions capable of facing the group’s main problems. Recent health emergencies, such as the Monkeypox outbreak in Brazil, revealed the need to expand gender identity and sexual orientation measurement, including this surveillance in all SINAN files, not only in the phenomenon of violence^(5)^. But what is perceived is precisely that, even when collected, the quality of data for these variables is relatively impaired, and their availability is restricted. Even after about 8 years of their inclusion in the violence surveillance form, these variables are still not accessible in SINAN’s public databases, limiting the carrying out of investigations and, consequently, public policy formation^(4)^.

Understanding multiple forms of violence from a broad perspective of gender identities is essential to combat inequalities and discrimination faced by self-perceived people within the trans spectrum. Understanding these manifestations and their structural causes is crucial to effectively and systematically dealing with the variety of violence faced by trans and non-binary people. Furthermore, a mechanism for epidemiological mapping of experiences of violence, which includes a variety of concepts about gender identities in studies and surveillance systems, would allow an objective, systematic and methodologically structured follow-up of the magnitude, characteristics and trends of the population phenomenon^(30)^.

It is important to emphasize that the indicators resulting from these measurement instruments would provide data to raise awareness in society about the seriousness of the problem, as well as support the development of more specific public policy formulations. A more accurate understanding of the patterns, characteristics, and factors associated with violence will make it possible to identify priority areas for intervention and develop strategies targeted at these population groups.

Therefore, it is essential and urgent to generate more sensitive approaches, both from an epidemiological and human rights perspective, that take into account the diversity of gender identities, allowing for the production of information to support policy makers. In addition to these gains, the review of procedures is likely to ensure information that enables spaces for the voices of people traditionally excluded and silenced by society and the state to be effectively heard. After all, are we facing a real process of inclusion, or could the process of recording gender identities on notification forms be merely an illusion?

## FINAL CONSIDERATIONS

Despite the indisputable advances with the introduction of the “gender identity” variable in the report form on interpersonal and self-inflicted violence in Brazil, it is fundamental that the limitations imposed on quality of information on gender in this form be frankly and urgently discussed. The adoption of a certain standard of gender normality, here ironically called “the originals” (women and cisgender men), introduces great uncertainties in the measurement process, as its identification is given by the combination of gender (male/female) with the response categories “ignored” and “does not apply”. From a theoretical and political point of view, this adoption reinforces a hegemonic power of language, in which there are right and wrong genders, reinforcing the discursive rupture between sex and gender as if one were something biological and, therefore, innate, and the other, a construction of roles and, consequently, an individual choice. Moreover, the way the form is presented also introduces important validity problems, ignoring other gender identities and producing estimation errors that can lead to wrong conclusions in the process of comparing groups for public policy formulation.

More than making criticisms without concrete propositions, in light of the reflections carried out, this essay intends to propose the urgent need to introduce the response categories “cisgender woman”, “cisgender man”, “intersex”, “gender fluid” and a field that allows open responses, such as “others”, ensuring the full right to gender self-determination. This, in addition to contributing to disruption of a long and hegemonic translation of sex as innate and gender as a social role, especially when dissonant from socially normalized rules, also tends to better gather information from reality, which is more complex than that proposed in binary and simplistic systems about gender. Ultimately, the report form would incorporate new nuances of gender identity, such as fluidity, intersex people and many other groups.
